# Supplementary material for: Publication bias in psychology: A closer look at the correlation between sample size and effect size
Source: PLoS One. 2024 Feb 15;19(2):e0297075. doi: 10.1371/journal.pone.0297075 (PMC10868788; doi:10.1371/journal.pone.0297075)
Supplement: Attachment — Submitted filename: ATJ response to R1 reviews final.docx [file pone.0297075.s001.docx]

*Dear reviewers, many thanks for your insightful and constructive feedback! We found it very useful to improve our paper as we detail below. (All our page numbers below refer to the printed page numbers on the clean copy of our revision.)*

Reviewer #1: The paper delves into the observed negative correlation between sample size and effect size in published reports within the field of psychology. It conducts two comprehensive studies to ascertain the underlying reasons for this correlation and to examine the impact of publication bias. In Study 1, the authors compare data from 150 meta-analyses across various subfields of psychology with 57 multiple replication datasets. They argue that because the replications are pre-registered, the chances of publication bias affecting them are lower. Based on their findings, the authors suggest that the small negative correlation between sample size and effect size is more likely the result of sampling adjustments, where researchers might be conducting smaller studies to capture larger effect sizes. Study 2 further scrutinizes this phenomenon by examining 160 randomly selected psychology papers. The authors find that focal effects (those central to the study’s hypothesis) tend to have larger negative correlations between sample size and effect size compared to randomly selected effects. However, these differences were not statistically significant. This leads to inconclusive evidence on whether publication bias has a differential impact on focal versus random effects. Furthermore, the negative correlation for random effects did not differ substantially from that found in meta-analyses, suggesting that publication bias might not be more pervasive in one versus the other. While the paper clarifies some aspects of the relationship between sample size and effect size, it leaves unanswered questions regarding the role of publication bias, particularly in focal versus random effects. The authors, recognizing these gaps, call for further research to better understand these nuances.
I find the authors’ approach to be commendable and thorough. The paper certainly contributes to our understanding of a complex issue, yet I find that the conclusions offer more questions than answers.
I also have concerns about the clarity of the paper, specifically with respect to the description of Study 1. The rationale for the different comparisons conducted is not clearly outlined, leaving me confused about which comparisons were pre-planned and which were post hoc. This lack of clarity makes it difficult to fully grasp the methodology and logic behind each comparison. For instance, the meaning of “cross-topics samples” and “within meta-analyses” does not become clear until much later in the paper from where these terms are first mentioned. The rational for the analyses is finally revealed in the discussion where the authors suddenly bring an important conclusion that there is little evidence of publication bias in psychology meta-analyses. It would have been much better to pre-empt this conclusion with an explanation in the methods detailing which results might support such a conclusion.

*We restructured the section that explains the aims of Study 1 (p7-9). We now provide an overview to guide readers (Table 2) and describe more clearly the characteristics of different study designs as well as the motivation behind specific comparisons. We also describe what conclusions would be supported by various results. Further, to aide our explanations, we moved Figure 2 forward. Finally, we revised results for Study 1 so that their order and terminology are better aligned with the description of our aims. We hope that these changes clear the earlier confusion.*

*Please note that Study 1 was not pre-registered. To make this point more salient, we now frequently use “explore”, “exploratory”, etc.*

Additionally, at the outset of Study 1, the reason for selecting meta-analyses is stated as the absence of sample size adjustments. Yet this seems to be the exact explanation the authors used to justify the small negative correlation found in Study 1.

*We regret the misunderstanding. We believe that the small negative correlation (within meta-analyses and within multiple replications) stems from the use of unsigned effect sizes (see Figure 2 and p13, middle). We hope that this point is clearer now.*

Please include Figure 1 in the next submission as it is missing from the manuscript.

*Apologies, this figure is now included.*

Additionally, I noticed that in Study 2, the agreement for converting effect sizes to Cohen's d between the two raters is surprisingly low (r=0.74). Given that this is supposed to be a formulaic calculation, this low level of agreement should have been discussed as a limitation in the paper.

*We agree that at first sight this could be seen as quite low. However, the differences did not arise from the calculation of d itself (which is indeed formulaic) but from identifying which of (typically multiple) analyses addresses the focal aim/hypothesis best. Despite several rounds of training, this remained a challenging issue. Kühberger et al. (2016) did not report relevant figures, which precludes a comparison with our reliability.*

In the general discussion section, the authors should discuss whether the correlation between effect size and sample size is indeed the most appropriate measure to use for this investigation. The authors advocate for this metric, but they do not thoroughly explore its limitations or consider alternative metrics that could also be informative.

*We now address this point in the general discussion (p24/25).*

In summary, while I appreciate the paper's aim and the rigor of its investigation, I believe it needs substantial revisions for clarity, discussion of limitations, and a more robust discussion of its key metric before it is ready for publication.

*Thanks again for your helpful comments!*

Reviewer #2: Review of Linden et al., ‘Publication bias in psychology: a closer look at the correlation between sample size and effect size’, submitted to PLoS ONE.

Summary

This study uses data from meta analyses and replication studies to investigate the correlation between sample size and effect size, which is typically negative. The results are obscured by a statistical artifact that is a consequence of taking absolute effect sizes, and are therefore difficult to interpret. It seems that this artefact is a consequence of using methods from previous studies, so I think there is potential for this paper to be reimagined as a critique of these methods, using the existing data and simulations. There are also some sloppy errors throughout, especially surrounding figures.

Specific points

1. The most serious issue I have with this work is that using the absolute effect size is almost guaranteed to introduce a spurious correlation in the direction reported. Since the authors use R, the problem can be illustrated by the following code, which simulates a series of random effect sizes and sample sizes centred on a true effect size of 0. By taking the absolute effect sizes, we introduce a strong negative correlation:

par(mfrow=c(1,2), las=1)

nstudies <- 200 # choose how many studies to include
trued <- 0 # set the true underlying effect size
truesd <- 1 # set the standard deviation of the underlying effects
alld <- NULL
allN <- NULL
\# simulate some studies using random sample sizes and effect sizes
for (n in 1:nstudies){
allN[n] <- 3+round(50*abs(rnorm(1)))
alld[n] <- mean(rnorm(allN[n],mean=trued,sd=truesd))
}

\# plot the signed effect sizes and calculate regression slope
plot(c(trued,trued),c(0,100),type='l',lwd=2,xlim=c(trued-truesd,trued+truesd),ylim=c(0,100),ann=FALSE,col=rgb(0.5,0.5,0.5))
points(alld,allN,pch=16,cex=0.5)
modout <- lm(alld ~ allN)
lines(modout$coefficients[1] + modout$coefficients[2]*(0:100),0:100,lwd=3,col='red')
title(xlab="Effect size (d)", col.lab=rgb(0,0,0), line=2.2, cex.lab=1.5) # titles for axes
title(ylab="Sample size (N)", col.lab=rgb(0,0,0), line=2.2, cex.lab=1.5)
title(main="Signed effect sizes", cex.main=1.5)

\# plot the unsigned effect sizes and calculate regression slope
plot(c(trued,trued),c(0,100),type='l',lwd=2,xlim=c(trued-truesd,trued+truesd),ylim=c(0,100),ann=FALSE,col=rgb(0.5,0.5,0.5))
points(abs(alld),allN,pch=16,cex=0.5)
modout <- lm(abs(alld) ~ allN)
lines(modout$coefficients[1] + modout$coefficients[2]*(0:100),0:100,lwd=3,col='red')
title(xlab="Effect size (d)", col.lab=rgb(0,0,0), line=2.2, cex.lab=1.5) # titles for axes
title(ylab="Sample size (N)", col.lab=rgb(0,0,0), line=2.2, cex.lab=1.5)
title(main="Unsigned effect sizes", cex.main=1.5)

The spurious correlation will become smaller as the mean effect departs from 0, which might explain some of the differences (i.e. between meta-analyses and replication studies) reported in the manuscript. It’s not clear to me if the authors are fully aware of this problem, even though it is very clear in Figure 2. There is a single mention of a statistical artifact on page 13, but it’s not explained in detail so I can’t tell if this is what’s being referred to or if it’s something else. I suspect this error is not the fault of the authors – it sounds like the convention of taking the absolute value has come from earlier studies, but it is clearly a huge problem. I would recommend repeating all analyses using the signed effect sizes, and using this comparison (as in Fig 2) as a critique of calculating the n-ES correlation using absolute values.

*Your simulation makes a similar point as our Figure 2, and we agree with it. Indeed, using signed effect sizes has the advantage that the expectation for the r_n-ES_ correlation is clearly zero in the absence of both PSB and sample-size adjustment. However, signed effect sizes have at least two disadvantages: i) They make meaningful comparisons with previous results (Kühberger et al.; Levine et al.) difficult. ii) Suddenly, not just negative but also positive n-ES correlations can indicate PSB. This is to be expected when the bulk of studies shows negative effect sizes. (E.g., flip the right panel in Figure 1 around the vertical axis and the n-ES correlation for the white studies changes to r = .59.) Moreover, although for unsigned effect sizes it is a priory unclear which r_n-ES_ correlation to expect in the absence of both PSB and sample-size adjustment, a sensible empirical comparison standard is available (i.e., the n-ES correlation within multiple replications). On the balance of arguments, we therefore decided to stick with unsigned effect sizes. However, following your suggestion, we added an analysis based on signed effect sizes within meta-analyses (p13, middle). Although statistically significantly smaller than zero, this correlation was very small (r_s_ = -.04) and our conclusions remain unchanged.*

2. Figure 1 is completely missing from the manuscript. On page 5 it says ‘see Fig. 1 [to be added]’.

*Apologies, the missing figure is now added.*

3. Plots of effect size against sample size are usually referred to as funnel plots. It’s worth using this terminology so that readers familiar with funnel plots realise that you’re talking about the same thing. Also, funnel plots are usually plotted with the effect size on the x-axis and the sample size on the y-axis. Is there a reason not to use this convention here (i.e. in Figs 2-4)?

*In the text, we now refer to Figure 1 as a funnel plot (p5), ditto in Figure 4. We also swapped axes in Figures 2-4 as suggested, so that precision is always on the y-axis and effect size on the x-axis.*

4. First paragraph – it’s not just PhD students that waste their time researching imaginary effects! I would change this to ‘and researchers might waste their time investigating’.

*Changed as suggested.*

5. Top of page 4 – it’s not clear why the correlation between N and effect size is ‘more suitable’, so this needs clarifying.
*We now write “More suitable [are] …, as we argue in detail below” (underlined = new addition). The detailed arguments follow on p4 (problems of alternative approaches) and p6 (how the n-ES correlation avoids these problems)*

6. Page 8 – the relevance of the sqrt(n) point about correlations is unclear and needs explaining.

*Our revised version omits this confusing and immaterial detail.*

7. I don’t buy the argument that meta-analyses are immune to sample-size adjustment. It’s usually the case that sample size estimates are conducted using effect sizes from previous relevant literature, so this must surely involve an influence of earlier studies on later ones. For example, if the first study reporting an effect has a huge effect size, the sample size of later studies will be informed by this (and will probably be small).

*We agree with your reasoning (e.g., we write “innocuous factors other than PSB can lead to a negative n-ES correlation. A particular concern is that researchers have some understanding of the magnitude of the effect they are studying and adjust their sample size accordingly, i.e., use larger samples to study small effects and smaller samples to study large effects”, p6). However, the researchers that produced the studies summarised in a meta-analysis work in the same field and therefore rely on the same available evidence about effect sizes. Hence, we elaborate that “researchers are unable to predict if their effect will turn out small, average, or large compared to other studies of the same topic” (p7, emphasis added). We hope that this point now becomes clearer in our revised version.*

8. Figure 2 is described backwards. The left panel is the abs values, but the caption says it’s the right panel. I think the two panels are just the wrong way around.

*The revised Figure 2 shows the panels in the correct order.*

9. Figure 4 is also described incorrectly. The text describes the within subjects studies as being small in sample size and large in effect size, which corresponds to the black crosses, but the figure caption says the within-subjects studies are the grey crosses. Also the two grey levels are quite hard to distinguish – consider using a different symbol for one condition.

*The revised Figure 4 should be easier to read and corrects the embarrassing error.*

*Thank you once more for your valuable comments!*
